# Evolution of Lactase Persistence: Turbo-Charging Adaptation in Growth Under the Selective Pressure of Maternal Mortality?

**DOI:** 10.3389/fphys.2021.696516

**Published:** 2021-08-23

**Authors:** Jonathan C. K. Wells, Emma Pomeroy, Jay T. Stock

**Affiliations:** ^1^Childhood Nutrition Research Centre, Population Policy and Practice Research and Teaching Department, UCL Great Ormond Street Institute of Child Health, London, United Kingdom; ^2^Department of Archaeology, University of Cambridge, Cambridge, United Kingdom; ^3^Department of Anthropology, University of Western Ontario, London, ON, Canada; ^4^Department of Archaeology, Max Planck Institute for the Science of Human History, Jena, Germany

**Keywords:** lactase persistence, origins of agriculture, niche construction, nutrition transition, childbirth, obstetrical dilemma, natural selection, milk

## Abstract

The emergence of the capacity to digest milk in some populations represents a landmark in human evolution, linking genetic change with a component of niche construction, namely dairying. Alleles promoting continued activity of the enzyme lactase through the life-course (lactase persistence) evolved in several global regions within the last 7,000 years. In some European regions, these alleles underwent rapid selection and must have profoundly affected fertility or mortality. Elsewhere, alleles spread more locally. However, the functional benefits underlying the rapid spread of lactase persistence remain unclear. Here, we set out the hypothesis that lactase persistence promoted skeletal growth, thereby offering a generic rapid solution to childbirth complications arising from exposure to ecological change, or to new environments through migration. Since reduced maternal growth and greater neonatal size both increase the risk of obstructed labour, any ecological exposure impacting these traits may increase maternal mortality risk. Over many generations, maternal skeletal dimensions could adapt to new ecological conditions through genetic change. However, this adaptive strategy would fail if ecological change was rapid, including through migration into new niches. We propose that the combination of consuming milk and lactase persistence could have reduced maternal mortality by promoting growth of the pelvis after weaning, while high calcium intake would reduce risk of pelvic deformities. Our conceptual framework provides locally relevant hypotheses to explain selection for lactase persistence in different global regions. For any given diet and individual genotype, the combination of lactase persistence and milk consumption would divert more energy to skeletal growth, either increasing pelvic dimensions or buffering them from worsening ecological conditions. The emergence of lactase persistence among dairying populations could have helped early European farmers adapt rapidly to northern latitudes, East African pastoralists adapt to sudden climate shifts to drier environments, and Near Eastern populations counteract secular declines in height associated with early agriculture. In each case, we assume that lactase persistence accelerated the timescale over which maternal skeletal dimensions could change, thus promoting both maternal and offspring survival. Where lactase persistence did not emerge, birth weight was constrained at lower levels, and this contributes to contemporary variability in diabetes risk.

## Introduction

The evolution of the capacity to digest milk in some populations appears a classic example of genetic adaptation in human evolution, made more interesting by the added ‘twist’ of niche construction (Odling-Smee et al., 2003). Lactase is an enzyme produced primarily in the small intestine that functions to break down lactose, the primary carbohydrate in mammalian milk, into glucose and galactose (Leonardi et al., 2012). Like other mammals, all humans are born with the ability to digest lactose, but by default this trait is lost around the age of weaning and the ancestral state for humans is for lactase to be down-regulated during childhood (Ingram et al., 2009a). Within the last 10,000 years, however, a number of human populations evolved a novel phenotype of lactase persistence (LP), where the gene remains active beyond mid-childhood. Roughly one third of contemporary human populations are therefore able to consume milk through the life-course without experiencing gastro-intestinal irritation (Ingram et al., 2009a). Among the majority who lose lactase activity, milk drinking tends to induce unpleasant or harmful symptoms, ranging from stomach cramps and bloating to severe diarrhoea, though there are examples of milk tolerance in populations lacking the alleles for LP (Lomer et al., 2008; Ingram et al., 2009a), suggesting that variable gut biota may provide an alternative metabolic tactic for digesting milk beyond infancy (Lukito et al., 2015).

Recent molecular analyses suggest that while alleles conferring LP spread rapidly in some global regions, the trait also evolved independently in several locations, and that more than one genetic mutation contributed, indicating an unusual degree of convergent evolution over a relatively short time-scale (Mulcare et al., 2004; Ingram et al., 2007; Tishkoff et al., 2007; Enattah et al., 2008). Within this global pattern, it is notable that the majority of lactose-tolerant humans are of European descent, and the underlying mutation spread fastest in this global region (Leonardi et al., 2012).

Crucially, alleles promoting LP could only realistically have undergone selection in populations whose diet included fresh milk from non-human animals, hence one way or another, the trait must have co-evolved with dairying (Leonardi et al., 2012). The two plausible scenarios are either that dairying spread rapidly among those who could digest lactose, or that LP underwent rapid selection amongst those practicing dairying.

Initially, anthropologists speculated that LP co-evolved with the agricultural custom of consuming the milk of domesticated animals – in particular cows, but also several other species such as goats and sheep (Simoons, 1970; Cavalli-Sforza, 1973). The earliest evidence for dairying dates to as early as 10,500 BP (Vigne, 2008) in South West Asia (Evershed et al., 2008), possibly South Asia by ∼8000 BP (Larson et al., 2014), Africa by ∼7000 BP (Bellwood, 2005) including the ‘green’ Sahara by ∼5000 BP (Dunne et al., 2012), and Southern Europe by ∼7200 BP (McClure et al., 2018), reaching Britain by 6100 BP (Copley et al., 2005). This geographical distribution could potentially have involved both cultural diffusion and the migration of farmers themselves. For example, there is growing evidence that European hunter-gatherers were not the ancestors of early farmers in the region (Bramanti et al., 2009), and that early farmers arrived from South West Asia, Anatolia and the Caucasus, before moving northwards into southern Scandinavia (Brandt et al., 2013). However, dairying very likely also emerged independently in different locations, as indicated by evidence for at least two domestication events for cattle, sheep and goats (Beja-Pereira et al., 2006).

More comprehensive studies indicate, however, that early farmers initially consumed milk products in forms where the lactose concentration was already reduced, such as cheese or yoghurt (Charlton et al., 2019). For example, early European farming sites are widely associated with a characteristic type of perforated pottery. Recent analysis has found the remains of milk fats on such pottery, indicating that it was used to separate fatty milk solids from liquid, lactose-rich whey (Salque et al., 2013). On this basis, early dairying could initially have provided a new source of nutrition without necessarily requiring the evolution of LP itself, moreover the archaeological record on dairying itself may not be easy to interpret, as dairy products might also have been stored in less preservable containers such as animal skins.

Prior to 8400 BP, all populations resident in Europe practiced hunting and gathering, yet by 6000 BP this subsistence mode had become rare and farming was dominant (Gerbault et al., 2011). Among these new farmers, the spread of LP appears to have occurred relatively rapidly, though also at variable rates across the region. Based on skeletal DNA analysis, early Neolithic European populations had low levels of LP (Burger et al., 2007; Malmstrom et al., 2010). A recent computer simulation suggested that the key allele first underwent selection among dairying farmers ∼7,500 years ago in central Europe (Itan et al., 2009), though this date has wide confidence intervals. From there, it appears to have spread rapidly among other central and northern European populations, associated with farmers replacing the local foraging populations in more northerly latitudes (Cramp et al., 2014a, b), though there is also evidence that climate-associated stresses slowed this northern advance (Betti et al., 2020). In contrast, the allele spread relatively weakly in southern Europe, and in this region the majority of contemporary individuals remain lactose-intolerant (Itan et al., 2010).

The spread of LP alleles in central and northern Europe was so rapid that it clearly had a major effect on the odds of survival and/or reproduction (Gerbault et al., 2011). According to one estimation, the mutation increased the fertility of carriers by around 15%, indicating a level of selection almost without precedent in relation to the human genome (Gerbault et al., 2011; Segurel and Bon, 2017). Lactase persistence clearly saved lives, or promoted fertility, but the underlying mechanisms remain unclear.

In some global regions, LP is closely associated with pastoralism, though this association is reconsidered below; and different populations can show markedly different prevalences of the trait depending on their subsistence history (Cox and Elliott, 1974; Swallow, 2003; Ingram et al., 2009a). Some have suggested that dairying only became possible among populations that had previously evolved LP (Vigne and Helmer, 2007), in other words that this metabolic trait drove the adoption of dairying. However, the broad consensus is that the existence of different genotypes for LP in different global regions makes this scenario highly unlikely; instead, it seems probable that subsistence patterns shaped the selective pressures acting on them (Odling-Smee et al., 2003; Laland et al., 2010), and that once dairying had emerged, it could provoke subsequent selection for LP. Consistent with that hypothesis, there is evidence from Turkey that milk was being consumed by populations that still have low frequencies of LP (Evershed et al., 2008), and that milk products were consumed in Britain before the likely emergence of LP (Charlton et al., 2019). Such studies indicate that the cultural practice of dairying preceded the emergence of LP, and that not all populations that started to practice dairying went on develop this metabolic trait.

A number of hypotheses have been proposed for the fitness value of LP. Building on the review by Gerbault et al. (2011), as well as more recent work by Wiley (2018) those hypotheses presented to date can summarized as follows:

• Arguably the simplest explanation is that milk is a rich source of dietary energy, and of protein and fat in particular. A cow can provide from 400 to 600 kg of milk per reproductive event, and could therefore contribute substantially more dietary energy per year than would have been obtained by consuming the same animal as meat (Beneke, 1994) (cited in Gerbault et al., 2011). Dairying could therefore have raised local energy productivity substantially, especially in marginal environments where humans have lower ability than stock animals to extract dietary energy from local plants.
• The energy supply from milk may have had heightened value during periods of famine, for example when cereal crops failed, an ever-present risk for early farmers (Gerbault et al., 2011). Children have greatest mortality risk during famine, as demonstrated by analysis of archeological skeletal collections (Herring et al., 1998; Temple, 2019) and recent demographic studies (Menken and Campbell, 1992), hence maintaining the ability to digest milk as a ‘fallback food’ through childhood and adolescence might have improved survival of these vulnerable groups.
• The ability to drink milk could have provided a source of uncontaminated fluid in arid environments (Cook and Al-Torki, 1975; Cook, 1978).
• Milk is an important dietary supply of calcium, essential for skeletal health, and also provides small quantities of vitamin D, which is less available through skin synthesis at higher latitudes. These benefits might have had added value among early farmers, consuming high-fibre diets with limited vitamin D content while probing more northerly territory (Flatz and Rotthauwe, 1973).
• Milk drinking could have conferred fitness benefits through the mediating role of elevated IGF1 levels on life history traits, such as increased body size and earlier maturation (Wiley, 2018).
• Milk consumption may protect against malaria by impeding folate synthesis, a key component of metabolism of the plasmodium parasite (Cordain et al., 2012).
• Milk drinking may have been a prestige activity (Simoons, 1970; Burger, 2010), hence if both the practice of drinking milk and the allele conferring LP were present among elites, any effects of natural selection could have been amplified by socio-economic factors (Durham, 1991; Heyer et al., 2005).

These hypotheses all remain relevant, nevertheless it remains difficult to see how they might have generated sufficiently strong selective pressures to account for the convergent evolution of lactose tolerance in different ecological settings, or its very rapid diffusion in Europe (Sverrisdottir et al., 2014).

Here, we propose and evaluate a different hypothesis: *that lactase persistence provided a solution to a generic problem for humans that emerged whenever exposure to local ecological factors constrained growth and/or favoured larger body size. In particular, changes in size and shape of the pelvis may increase both maternal and perinatal mortality, hence any factor that could accelerate the amelioration of this challenge should be under strong selection*. Since consuming milk may promote both overall growth of the pelvis and a more favourable shape of the birth canal, milk-drinking populations in different global regions might share an accelerated relaxation of selective pressures acting on maternal mortality.

Our hypothesis thus proposes a fundamental link between a ‘hallmark genetic adaptation’ of human evolution on the one hand, and a ‘hallmark anatomical adaptation’ (the obstetric pelvis) on the other. Moreover, this approach might also help explain why LP evolved several times in different ecological settings through selection for different alleles. Finally, we hypothesise that *where lactase persistence did not evolve, birth weight remained low, and may have decreased in association with downwards trends in stature*, patterns of growth that now contribute to an elevated risk non-communicable disease in many populations as they undergo modernization and ‘nutrition transition’ (Popkin et al., 2020).

## The Genetic Basis of Lactase Persistence

Lactase persistence is an autosomal dominant trait. Studies of its prevalence have been undertaken since the 1960s, using several different tests. Most of these involve giving an oral lactose load, and then testing for either changes in blood glucose content, an increase of galactose levels in urine, breath hydrogen production, or the severity of intestinal symptoms (Segurel and Bon, 2017). Integrating data from these tests, recent research has shed substantial new light on the global distribution of alleles for LP, though the picture remains incomplete (Segurel and Bon, 2017).

In Europeans, LP is strongly associated with a single C to T mutation 13,910 bp upstream of the lactase (LCT) gene (–13,910^∗^T) (Segurel and Bon, 2017). Recent studies have shown that this variant also accounts for the vast majority of the LP phenotype across Eurasia, including Russia, Iran and the Indian subcontinent (Enattah et al., 2007; Heyer et al., 2011; Gallego Romero et al., 2012). It is also the primary mutation associated with LP in some north and sub-Saharan African populations (Ingram et al., 2007; Ranciaro et al., 2014).

**FIGURE 1:**
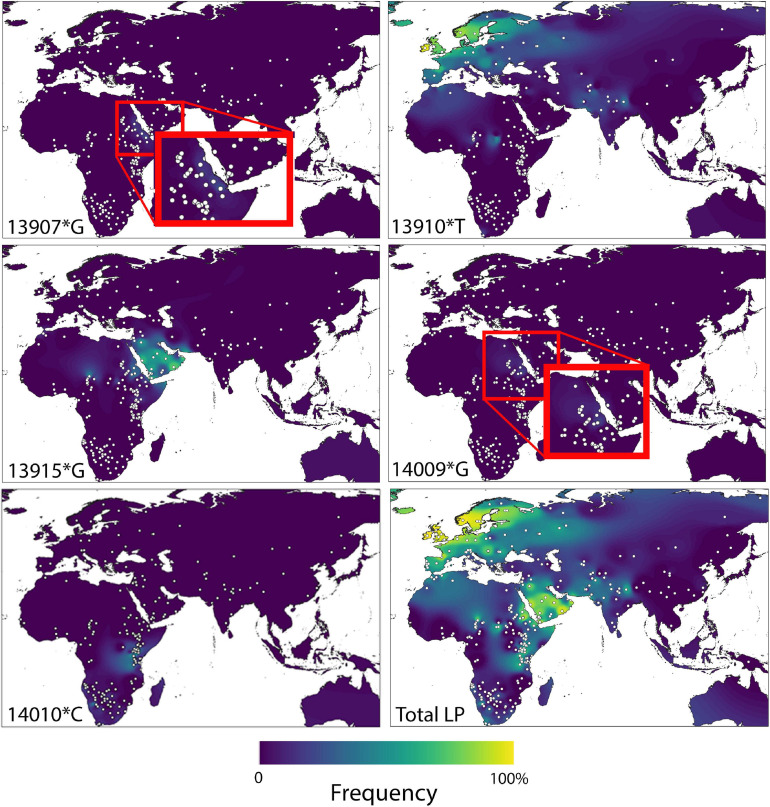
Geographic distributions of LP causative variants –13910^∗^T, –13915^∗^G, –13907^∗^G, –14010^∗^G, and –140101^∗^C in the old world, as well as composite lactase persistence. Reproduced with permission from Liebert et al. (2017).

This monogenic basis of LP in Eurasia contrasts however with the situation in sub-Saharan Africa, where an additional four alleles (–13.915:G; –13.907:G; –14.009:G; –14.010:C) have so far been associated with the phenotype (Mulcare et al., 2004; Ingram et al., 2007, 2009b; Tishkoff et al., 2007; Itan et al., 2010), and further mutations might also contribute. The main geographical distribution of these alleles is summarized in Figure 1, along with a composite global map displaying total LP prevalence.

Additional studies have shown that LP has undergone relatively strong selection in recent time periods (Field et al., 2016). The rate of selection is considered rapid in both Eurasian and African settings. Compared to other human loci that are also considered to have undergone strong selection (e.g., malaria resistance, skin pigmentation in Europeans, adaptation to hypoxia, alcohol metabolism), LP appears to demonstrate an especially strong signal of selection (Segurel and Bon, 2017). This challenges interpretation of any putative selective advantages in groups with low frequencies of LP, as this may simply indicate admixture with neighbouring groups with high frequencies (Segurel and Bon, 2017).

Nevertheless, this genetic epidemiology also reveals some puzzling scenarios. For example, there are several populations of herders from Mongolia and East Asia who drink milk but have low frequencies of LP alleles (Segurel and Bon, 2017); conversely, there are several populations of foragers in eastern and southern Africa who have high frequencies of LP alleles, despite not drinking milk. Moreover, some European populations only demonstrated high frequencies of LP alleles relatively recently in the medieval ages, despite milk drinking having been practiced for much longer periods (Segurel and Bon, 2017). Current genetic data do not therefore perform very well in explaining global phenotypic diversity, and additional genetic investigations are warranted (Itan et al., 2010).

Despite the evidence gaps described above, it is already clear that the phenotype of LP has emerged through different forms of genetic change, and therefore represents a striking example of convergent evolution, which in turn strongly implicates natural selection. Conventionally, such convergent evolution is considered a challenge to many of the hypotheses for the fitness benefits of LP, as most attribute its benefits to ecological factors that are quite specific to a given geographical region. Our hypothesis represents a solution to this ‘heterogeneity’ challenge. On the one hand, maternal mortality during childbirth is potentially a stress for all human populations, on the other hand the proximate environmental challenges, and the associated human adaptations that help resolve those challenges, may vary substantially across geographical regions and populations.

## The Obstetrical Dilemma

The evolutionary significance of variability in skeletal dimensions for childbirth has long been recognized. Among the key characteristics that emerged during the evolution of our genus were bipedal locomotion and increased brain size (Rosenberg, 1992). Building on the insight that the greater width of the female pelvis relative to that of males serves ‘the function of a birth canal’ (Nicholson and Allen, 1946), the anthropologist Wilton Krogman proposed that encephalisation had made childbirth substantially more problematic in *Homo*, given that the maternal pelvis had also undergone changes to support bipedal locomotion that had narrowed the birth canal. Sherwood Washburn described this interaction as an ‘obstetrical dilemma’, resulting from antagonistic selective pressures favouring bipedality and fetal encephalisation (Washburn, 1960). To resolve this problematic interaction, humans have evolved a complex rotational birth mechanism, and are unusual in the near-universal tendency for women to seek assistance during delivery (Rosenberg and Trevathan, 2002), though there are occasional records of individual women delivering unaided (Shostak, 1981).

Recently, Washburn’s notion of antagonistic selective pressures has been critiqued. Maternal pelvic dimensions vary substantially within and across populations with no obvious impact on maternal locomotor biomechanics (Warrener et al., 2015). There must therefore be other factors that account for variability in the size and shape of the birth canal, in order to explain why obstructed labour remains a major health burden in many contemporary populations (Ronsmans et al., 2006).

From a genetic perspective, delivery can be characterized by the interaction of two contrasting fitness functions. The discrepancy between pelvic and neonatal dimensions of individual mother-offspring dyads demonstrates a normal distribution; in contrast, individual female fitness demonstrates a ‘cliff-edge’ form, because delivery changes from being possible to impossible as soon as the size of the fetus exceeds a certain threshold (Mitteroecker et al., 2016). On this basis, it has been suggested that the phenotypic distribution that maximizes population mean fitness will inevitably be associated with a proportion of individuals exceeding the ‘cliff-edge,’ where the baby is too large for natural delivery (Mitteroecker et al., 2016). However, like the original obstetrical dilemma hypothesis, this argument does not explain population variability in the incidence of obstructed labour.

**FIGURE 2:**
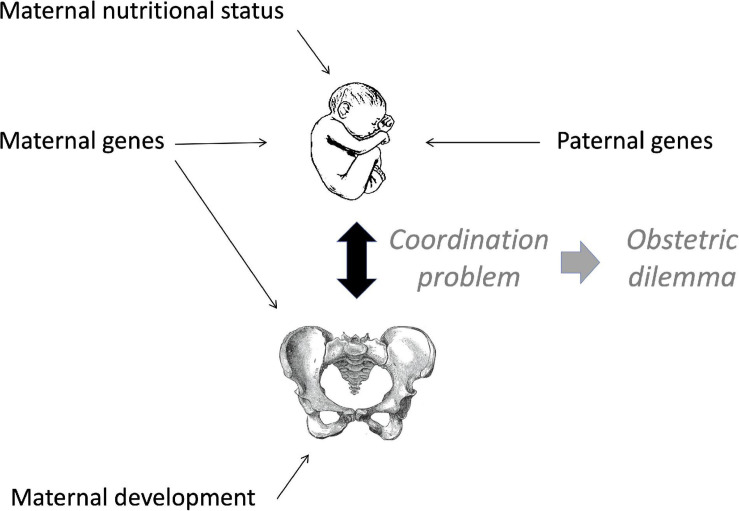
Schematic diagram of the coordination problem. Adapted from Wells (2015).

An alternative evolutionary perspective considers that the obstetrical dilemma is not a fixed human trait, but rather the outcome of a ‘coordination problem’ between the dimensions of the maternal pelvis and the fetus at the time of birth, which may also reflect paternal genotype (Figure 2; Wells, 2015). On this basis, the obstetrical dilemma may vary within populations and across generations on account of variability in either maternal or offspring somatic dimensions (Wells et al., 2012). Factors that reduce maternal pelvic dimensions, or that increase neonatal size, would both be expected to exacerbate the obstetrical dilemma through ‘poor coordination’, increasing the risk of obstructed labour. This perspective is consistent with the idea that ‘the female pelvis is by far the most important part of the skeleton in terms of ‘fitness’ for producing offspring’ (Vieth, 2020).

In turn, at the proximate level, variability in maternal pelvic dimensions and neonatal size may initially emerge via adaptive responses to ecological stimuli or stresses, though these may act over different timescales within the life-course and across generations (Wells et al., 2012). For example, maternal growth variability may emerge in response to local ecological conditions, while fetal variability may emerge in response to maternal metabolic phenotype during pregnancy (Wells, 2010, 2017; Wells et al., 2018). Diverse ecological factors may thereby shape both maternal growth and fetal growth, and hence ultimately impact the ‘coordination problem’. That the coordination problem is variable at the level of individual mothers is supported by evidence that paternal phenotype can predict the risk of childbirth complications. For example, taller paternal height (a marker of greater fetal growth drive) increases risk of caesarean delivery (Stulp et al., 2011), while mixed-ethnic unions (e.g., South Asian mothers and European fathers) show a similar elevated risk relative to two parents of the same ethnicity (Nystrom et al., 2008), mediated by contrasts in maternal and neonatal size (Wells et al., 2013).

Any variability in maternal or fetal dimensions may require the phenotypic coordination between the two parties to be ‘renegotiated’ (Wells, 2015). In the long-term, this may arise through genetic change in either maternal or fetal phenotype, under the influence of selection. Of relevance here, Grabowski argued that divergent selection pressures may have reduced the magnitude of constraint on birth canal evolution, in turn reducing the time required for evolutionary change (Grabowski, 2013). This could facilitate the adaptation of birth-associated morphology to new niches, or to secular change in ecological conditions. Recent work has also clarified that the obstetric and locomotor pelvis are morphologically distinct, with the obstetric pelvis showing greater flexibility (Ricklan et al., 2021).

Within the life-course, such ‘renegotiation’ could also take place through plastic mechanisms, for example where maternal growth reflects ecological conditions (Ricklan et al., 2021), or where fetal growth responds to signals of maternal metabolism (Wells, 2015). Birth weight appears to have a relatively weak genetic basis (Yokoyama et al., 2018), and this is expected, because if it were strong there would be a very high risk of childbirth complications whenever mothers had failed to reach their growth potential (Wells, 2015). On this basis, fetal growth should primarily be shaped by signals of maternal metabolism during pregnancy. This hypothesis is broadly supported, but perturbations in maternal metabolism associated with overweight and obesity appear to disrupt the regulatory system (Wells, 2017).

In the following discussion, we consider population differences in growth traits. Despite underlying genetic variability that influences size, we review the importance of plastic responses in growth, as the role of plasticity in the obstetrical dilemma has also been demonstrated (Wells et al., 2018). We focus on maternal traits since the key effect of LP is on biology from childhood onwards, though we also consider potential influences on the fetus through the mediation of maternal metabolism during pregnancy.

### Maternal Growth and Reproductive Fitness

All other things being equal, larger maternal size tends to be beneficial for fitness, though more than one underlying pathway is relevant. One key pathway acts through maternal skeletal dimensions. Numerous studies from every global region have shown that shorter mothers have increased risk of caesarean section (Mahmood et al., 1988; Sokal et al., 1991; Amoa et al., 1997; Merchant et al., 2001; Seshadri and Mukherjee, 2005; Toh-Adam et al., 2012; Wells, 2017). This association can be attributed in part to a greater risk of cephalo-pelvic disproportion and other forms of obstructed labour (Wells et al., 2021). Obstructed labour threatens both mothers and infants, and still accounts for ∼12% of global maternal mortality (World Health Organisation, 2005, 2006). Although the threshold of risk varies by geographical region (Wells, 2017), the link between short stature and childbirth complications appears to be a human universal.

Regarding mechanism, the association of maternal height with obstructed labour appears to be primarily mediated by maternal pelvic dimensions. At the population level, taller mothers have larger pelvic dimensions (Table 1), decreasing susceptibility to obstructed labour. Across two generations, secular increases in height were associated both with increasing pelvic dimensions and with reduced risk of cephalo-pelvic disproportion (Holland et al., 1982).

**TABLE 1:** Associations of maternal height and pelvic dimensions.

Population	*n*	Association	References
Ghana	113	Correlation of height (H) and true conjugate TC *r* = 0.51, *p* = 0.0001 Group comparison for mean H and TC H = 149.6, TC = 9.6; H = 157.1, TC = 10.2; H = 164.1 TC = 10.8 cm	Adadevoh et al., 1989
Nigeria	612	Frequency of contracted pelvis inversely associated with maternal H	Cox, 1963
Rwanda	152	Frequency of contracted pelvis inversely associated with maternal H	Kakoma, 2016
India	197	Pelvic area (PA) correlated with height (H), *r* = 0.31, *p* < 0.01 Group comparison: *H* > 150 cm, PA = 30,516 mm^2^; *H* = 150–160 cm, PA = 31,820 mm^2^; *H* > 160 cm, PA = 34,103 mm^2^	Sharma et al., 2016
Scotland	200	Tall women have larger brim index and sagittal index than short women	Bernard, 1952
United Kingdom	68	Maternal height and leg length associated with internal and external dimensions of pelvis in South Asian nulliparous women	Shirley et al., 2020
Austria	1977	Tall women (*n* = 1870, *H* = 163.8 cm) had larger external conjugate (EC = 20.2 cm) than short women (*n* = 107, *H* = 158.4, EC = 19.6), *p* < 0.05	Hanzal et al., 1993

*H, height; TC, true conjugate; EC, external conjugate; P, pelvic area; T, transverse; AP, anterior-posterior.*

**FIGURE 3:**
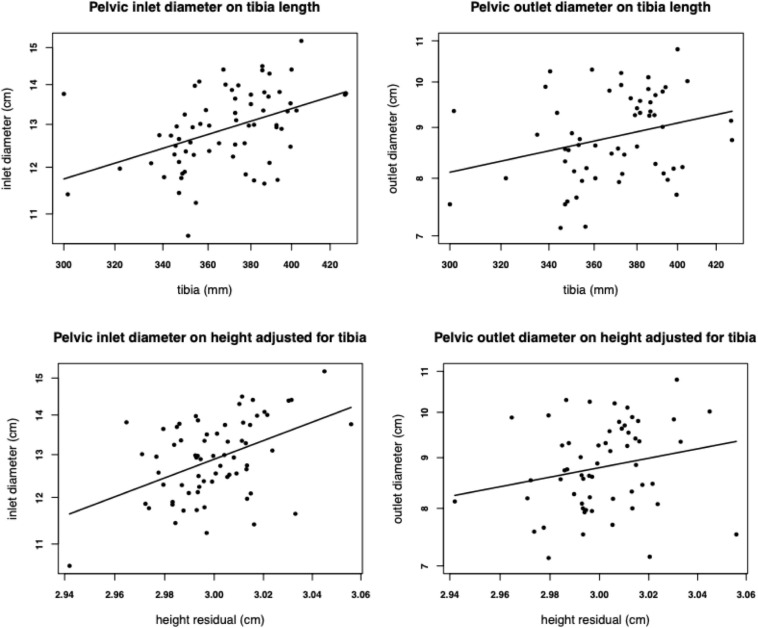
Associations of tibia length, and the component of height variability that is statistically independent of tibia length, with the diameter of the pelvic inlet (*n* = 67) and outlet (*n* = 58) in nulliparous young adult women of South Asia ancestry living in the United Kingdom. Reproduced with permission from Shirley et al. (2020).

Among nulliparous South Asian women living in the United Kingdom, height correlated positively with six pelvic dimensions (Shirley et al., 2020). When height was subdivided into two components of variability (tibia length, and another component statistically independent of tibia length), both components predicted pelvic dimensions relatively equally (Figure 3). Since tibia length is particularly sensitive to environmental conditions in early life (Gunnell et al., 1998), whereas the other component reflects conditions throughout development and genotype, the implication is that growth during any period or through any mechanism promotes larger pelvic dimensions. Significant for our argument, variability in leg length is the primary mediator between childhood dietary intake and adult stature (Gunnell et al., 2002).

A second key pathway acts through maternal body size more generally, as this benefits birth weight which in turn is a key determinant of offspring survival (Hogue et al., 1987). Although maternal body fat funds post-natal infant growth via lactation, during pregnancy the primary determinants of fetal growth are maternal metabolic turnover and its correlate, fat-free mass (Martin, 1983; Wells, 2018b). Mothers with greater fat-free mass and energy turnover pass a greater absolute quantity of nutrients across the placenta (Martin, 1983; Wells, 2018b). Again, maternal growth is a key mediator, as height is a primary determinant of both fat-free mass and resting metabolic rate (Lee et al., 2000; Frankenfield et al., 2005). However, the pelvis may also contribute to these associations, for example an analysis of Brazilian mothers found that associations of maternal BMI with offspring birth weight were mediated by pelvic dimensions (Wells et al., 2017).

In combination, these associations contribute to significant fitness penalties experienced by shorter/smaller mothers and their offspring. Across 54 low- and middle-income countries, for example, shorter maternal height was associated with increased rates of wasting, stunting and child mortality among the offspring (Ozaltin et al., 2010). From an evolutionary perspective, the fitness penalties of maternal mortality are substantially greater than those of infant mortality, as the death of an infant may rapidly be compensated by the mother conceiving again, whereas maternal death clearly allows no such response. The potential fitness penalties associated with inadequate maternal growth are of particular relevance to our aim of suggesting why LP alleles spread so rapidly in a few thousand years.

However, it should be noted that other factors may favour smaller maternal size. In particular, a higher extrinsic mortality risk favours earlier maturation, in order to reduce the chance that mortality occurs before reproduction (Walker et al., 2006). Similarly, a chronically low energy supply may also favour smaller adult size, in order to reduce the time required for maturation (Walker et al., 2006; Kramer and Greaves, 2010). In any ecological setting, therefore, maternal skeletal growth emerges as a compromise in relation to competing selective pressures. This issue is revisited below, in relation to environmental temperature, however, the relationship between size and pelvic dimensions can itself adapt over time. For example, a late Palaeolithic population, whose smaller body size appears an adaptation to stable ecological conditions, evolved larger pelvic dimensions than might be expected given their short stature, suggesting selection on easier delivery (Kurki, 2007).

## Milk and Growth

We now consider how the subsistence mode of dairying, and in particular the consumption of milk itself, may have influenced human growth and metabolism. That mammalian milk is a growth-promoting substance is self-evident. Even though the precursor to lactation may have originally evolved in the form of secretions to boost offspring immunity (Oftedal, 2012), this form of nutrition supports the growth of all mammalian offspring between birth and weaning. However, milk stimulates growth in more ways than simply providing energy, protein and micronutrients. Lactation also ‘protects’ growth from pathogens by reducing the risk of infection, and by containing a variety of immune agents that prime the infant immune system (Cacho and Lawrence, 2017). Every mammalian milk can be considered a complete and sophisticated endocrine system, shaped by the selective pressures acting on that species (Melnik et al., 2015), furthermore lactation is not a uniform passive process but a dynamic interactive one, through which mother and offspring can communicate through physiological and behavioural signalling mechanisms (Wells, 2018a; Mohd Shukri et al., 2019; Fewtrell et al., 2020).

The importance of growth-promoting hormones becomes clearer, as soon as we focus on the impact of consuming the milk of other species after breast-feeding itself has ceased. Cows’ milk is not only high in protein, but is also a natural source of two proteins that stimulate linear growth, namely growth hormone and insulin-like growth factor I (IGF1) (Hoppe et al., 2006). At a more fundamental level, milk also stimulates mTORC1 signalling, which plays a key nutrient-sensitive role in regulating growth and metabolism (Melnik et al., 2015). Observational, ecological and experimental studies all provide evidence that animal milk consumption promotes human growth.

### Observational Studies

Observational evidence from multiple sources broadly indicates that throughout the period of development, consuming animal milk promotes human growth. In infancy, the use of cows’ milk formula affects growth primarily by increasing weight gain, but a few studies found that linear growth was also greater in formula-fed infants in later infancy (Michaelsen et al., 1994; Dewey et al., 1995; Nielsen et al., 1998). In preschool children, observational studies from Europe, South America, Asia and sub-Saharan Africa have linked milk intake with greater linear growth (Ruel, 2003; Hoppe et al., 2004; Mosites et al., 2017; Choudhury and Headey, 2018; Wiley et al., 2018; Miller et al., 2020; Vanderhout and Corsi, 2020).

Among older children and adolescents, several observational studies have likewise linked milk consumption with greater linear growth (Hoppe et al., 2006; Dror and Allen, 2014). In the US NHANES 1999–2002 dataset, for example, childhood milk consumption was positively associated with adult height (Wiley, 2005), however, separating the effects of milk consumption in different periods showed a cross-sectional association of milk intake and height among at 12–18 years but not at 5–11 years (Wiley, 2005). However, a longitudinal study from the same country showed no difference in the association of milk consumption with growth by developmental period, rather for each additional 236 mL of milk consumed per day throughout childhood and adolescence, height was 0.4 cm greater (Marshall et al., 2018).

These studies are supported by historical research. Local milk production was associated with greater height in adult men in the 19th century in several regions of France and Germany (Baten, 2009). Similarly, high milk intake is considered to have played an important role in the maximal secular trend in height demonstrated by the Netherlands population over the last two centuries, while increased milk intake also appears to have contributed to secular increases in growth between 1950 and 1990 in Japanese children (Takahashi, 1984).

### Ecological Studies

Among pastoralists, milk often comprises a substantial proportion of daily energy intake, and in some East African pastoralist groups such as the Turkana, Rendille, Maasai, and Borana, this may exceed 50% of total energy intake (Galvin, 1992; Lindtjorn et al., 1993; Galvin et al., 1994; Homewood, 1995). Pastoralist populations tend have higher adult stature in comparison with neighbouring populations practicing cultivation (Takahashi, 1971; Guntupalli and Baten, 2006), and this has been attributed to the significant contribution of dairy products to total dietary protein and energy intake, promoting child growth (Little et al., 1983; Little and Johnson, 1987). In a national survey of anthropometric data from India, pastoralists likewise showed relatively tall stature despite being of low rank in the caste system, due to their priority access to milk products (Guntupalli and Baten, 2006). Abandoning pastoralism in association with a shift to sedentary crop-farming has been linked with detrimental effects on child growth. For example, comparing settled communities of Rendille in northern Kenya with those still practicing pastoralism, children from settled communities were shorter, attributed in part to their reduced milk consumption (Fratkin et al., 2004).

### Interventions

**FIGURE 4:**
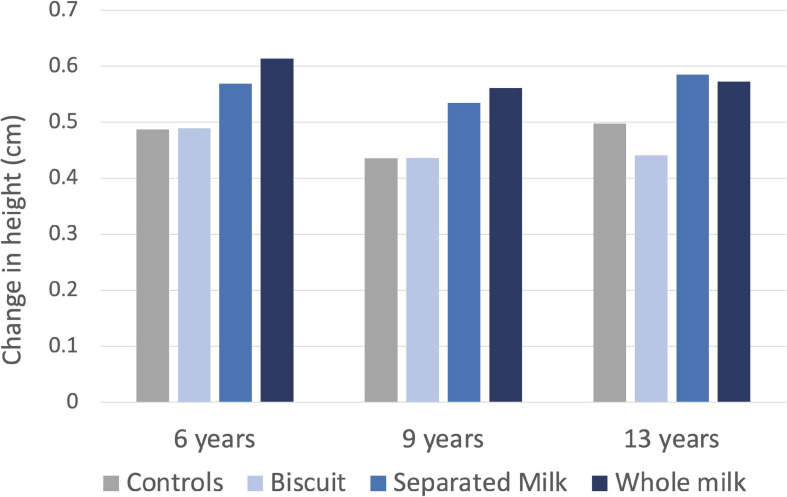
Association of nutritional supplementation with linear growth in Scottish children from 3 age groups in the 1920s. Children given either whole milk, skimmed milk, or biscuits containing the same amount of energy were compared to an unsupplemented control group. Data from Orr (1928).

The strongest evidence derives from interventions studies that have tested the effects on growth of supplementation with milk. These studies have generally associated increased milk intake with greater growth, especially among populations that are generally subject to low protein intakes. For example, in 1926-7 Boyd Orr assessed the effects of supplementing the diet of children aged 6–13 years in several Scottish cities with either whole milk, skimmed milk, or biscuits containing the same amount of energy, compared to an unsupplemented control group. Irrespective of their age, the children who received either form of milk supplement grew ∼20% more in height over 7 months compared to the controls, whereas those given the biscuits showed no difference (Figure 4; Orr, 1928).

Similarly, among children aged 7–13 years in Papua New Guinea over a 13-week period, those given a supplement containing skim milk powder showed a growth velocity twice that of other groups given supplements without milk (Malcolm, 1970; Lampl et al., 1978). In a study of 10-year-old girls in Beijing, a supplement of milk (without or without cholecalciferol) significantly increased linear growth (Du et al., 2004). In India, children who received a 1-year milk-protein and micronutrient-fortified food product showed improved linear growth compared to those with lower nutrient intakes (Thomas et al., 2020). However, an intervention in Guatemala that provided a supplement containing skim-milk protein did not promote growth compared to a supplement containing energy only (Martorell and Klein, 1980).

While these experimental studies are relatively consistent, in showing that drinking milk promotes growth, they are less so for the broader category of dairy products, which includes foods such as yoghurt and cheese. A review of 12 randomized control trials interventions involving dairy products found that their consumption was associated with an increased rate of growth of 0.4 cm/year, for each additional 245 ml of milk-equivalent consumed daily, but the available evidence was not considered of high quality (De Beer, 2012). A more recent review of 13 interventions found the evidence to be inconclusive, with six trials showing a positive association and seven finding no association (De Lamas et al., 2019). However, this heterogeneity may partly arise from combining both milk drinking and dairy consumption into a single exposure, as the prior meta-analysis indicated that the effect on height of drinking milk itself is stronger than that of consuming dairy foods (De Beer, 2012).

### Mechanisms

The underlying mechanisms linking milk with growth during different periods of development are still being elucidated. While some studies have linked milk consumption with higher levels of IGF1 in both children and adults, others have shown null associations (Hoppe et al., 2006). One possibility is that the IGF1 axis itself is programmed by milk intake during early life, making that period a ‘critical window’ that might shape the impact of milk consumption at later ages (Hoppe et al., 2006). Other relevant pathways may involve stimulation of the insulin response, growth hormone, bioactive peptides, amino acids, milk minerals, and mTORC1 signalling (Hoppe et al., 2006; Melnik et al., 2013). For example, a study of Mongolian children aged 10–11 years found that levels of both IGF and growth hormone increased after a month of elevated milk intake (Rich-Edwards et al., 2007), while a study of calcium supplementation in pre-pubertal girls over 1 year found that stature increased as well as bone mineral density, especially in those whose spontaneous calcium intake was below the median level (Bonjour et al., 1997).

Whether or not milk promotes growth may of course also depend on the level of lactase activity in the population. A study of Swedish adolescents demonstrated that both milk consumption and LP independently promote height. Adjusting for parental height, birth weight, socio-economic status, sex and BMI as an index of nutritional status, height increased by 0.45 cm per quintile of milk consumption, and was 2.0 cm greater among those with LP (Almon et al., 2011). However, several other studies have reported no simple association between LP and height (Lehtimäki et al., 2006; Corella et al., 2011), hence the respective contributions of LP genotype and exposure to milk require further research.

### Milk and Growth of the Pelvis

In the vast majority of previous growth studies, the primary outcome has been either height or linear growth rate, and few studies have considered more specific skeletal dimensions related to childbirth. In the only relevant study to date, on 326 women aged 45–60 years from Hawaii, milk intake during adolescence was found to be associated with an external dimension that included the pelvis (the horizontal distance between the outermost points of the greater trochanters of the femora), but not with two markers of internal canal dimensions (Novotny et al., 2000). However, this study did not test whether milk consumption was associated with adult height.

More generally, however, there is growing evidence that consuming milk affects a wider range of traits than skeletal growth alone. From a life history perspective, there is some evidence that milk consumption may promote earlier sexual maturation (Wiley, 2012), and this too could have fitness implications by shortening inter-generation intervals. Given widespread associations of height with more favourable childbirth outcomes, and emerging mechanistic evidence that height is positively associated with dimension of the obstetric pelvis, we assume that drinking milk would benefit childbirth complications in part through promoting the size of the obstetric pelvis, however, further evidence is required to support this hypothesis more robustly.

### Maternal Metabolism and Nutritional Status

It should be noted, however, that consuming milk might not only effect linear growth, potentially promoting maternal height and pelvic dimensions, but might potentially also affect maternal metabolism, influencing body mass, energy stores and metabolic homeostasis. These are also pathways that could impact maternal fitness.

In high-income countries, milk is currently made commercially available with different levels of fat content, such that the total energy content of full-fat milk (∼266 kJ per 100 ml) is substantially greater than that of skimmed milk (147 kJ per 100 ml). However, in the past, milk would universally have represented a source of nutrition relatively high in energy, fat and protein, and it might be assumed that it would therefore promote maternal body fatness. Recent studies have also associated LP itself with higher BMI (Kettunen et al., 2010; Corella et al., 2011; Montalva et al., 2019), and this effect is stronger among those who consume higher levels of dairy products (Lamri et al., 2013). However, there is also emerging evidence that the milk of some species is associated with lower diabetes risk, suggesting that milk consumption might influence maternal glycaemic control (Agrawal et al., 2007, 2011).

### Summary of the Effects of Milk on Growth

Overall, milk appears to stimulate both linear growth and relative weight of mothers. Both pathways could benefit maternal fitness, by reducing maternal mortality risk through reducing the risk of obstructed labour, and increasing offspring birth weight and hence survival. However, since greater birth weight may also exacerbate childbirth complications, the net effect of milk consumption on delivery merits consideration, as addressed below.

We suggest that the key evolutionary significance of LP at a mechanistic level is not directly through effects on size, but rather the speed with which skeletal dimensions can change. Even under strong selective pressure, ‘genetic resolution’ of the obstetric dilemma may require many tens or hundreds of generations, whereas the single allele of LP has a very different effect – eliciting greater skeletal growth in the absence of any direct change in the frequency of individual alleles for height. Drinking milk in association with LP essentially ‘turbo-charges’ growth, efficiently allocating more energy to growth traits instead of to other life history functions (Hill, 1993). In turn, this could accelerate the adaptative response of growth traits to any change in ecological conditions.

#### Milk and the Prevention of Pelvic Deformity

Beyond associations of maternal short stature with smaller pelvic dimensions, specific nutritional deficits are also important. The best-known example is rickets, widely established to be a risk factor for poor pelvic development (Nicholson and Allen, 1946; Skippen, 2009).

**FIGURE 5:**
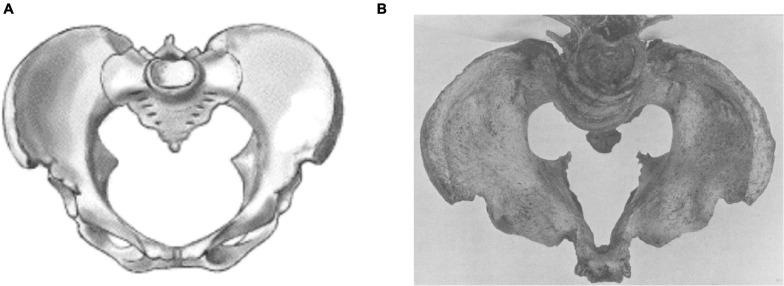
Comparison of shape between **(A)** a normal pelvis and **(B)** one affected by rickets. Reproduced with permission from Maxwell (1935) and Vieth (2020).

Early 20th century obstetricians noted high perinatal mortality in the main urban centres of 19th century industrialization (Baird, 1949; Illsley, 1966), and linked this with maternal short stature. However, a large proportion of the resulting childbirth complications could be specifically attributed to rickets, which involved significant flattening of the pelvis (Figure 5) in response to nutritional deficiencies during early maternal development (Dick, 1922).

In contemporary populations, the primary pathway to rickets is through Vitamin D deficiency, resulting from inadequate diet or insufficient exposure to sunlight. Intriguingly, Vitamin D deficiency has been associated with increased risk of caesarean delivery (Merewood et al., 2009). Although several mechanisms may contribute, the historical evidence reviewed above suggests that pelvic constraint contributes. Populations in northern latitudes, exposed to seasonal fluctuations in endogenous vitamin D synthesis, may be at increased risk of rickets. Specifically, infants born in winter may experience constraint on early pelvic growth, with long-term effects on the risk of childbirth complications in adulthood (Strickland, 1993). This might explain high, and seasonally variant, rates of maternal mortality in Mongolia, a population characterized by high prevalences of maternal short stature and rickets: higher levels of fetal growth may tip individual mothers over the threshold for childbirth complications, if they had previously experienced poor nutrition in early life (Strickland, 1993).

Though a challenge in recent historical period, rickets is generally presumed to have been very rare before the second millennium AD (Wells, 1975), and hence not to have been a major evolutionary stress. However, this assumption merits re-appraisal: although rickets is widely associated with inadequate vitamin D levels, and tends to emerge during the first year of life, there is also a form that develops after 1 year of age despite adequate sunshine exposure (Thacher, 2003). The cause of this later-onset form appears to be inadequate dietary calcium. Although reports to date come largely from Bangladesh, Nigeria and South Africa, it is possible that the condition is more widespread in low- and middle-income countries, and it has also occasionally been observed in vulnerable groups in high-income countries (Thacher, 2003). Moreover, rickets can also develop during adolescence, again in association with dietary constraints (Hunter et al., 1984).

While animal milks make negligible contribution to vitamin D requirement, they are undoubtedly rich in calcium (Barłowska et al., 2011; Claeys et al., 2014), and could therefore help to prevent calcium-deficiency rickets. Indeed, animal milk products are generally the primary source of dietary calcium after weaning. According to recommended daily intakes for European populations, 100 ml of the milk of most domesticated species would either fully or almost meet daily adult calcium requirements (Claeys et al., 2014). More generally, the milks of all domesticated species are rich not only in minerals essential for bone mineralization (calcium, phosphorus) but also energy and protein (Table 2), though there are few data on Vitamin D content. However, since milk products such as yoghurt and cheese also provide calcium, this pathway may only be relevant to selective pressures on maternal mortality if pelvic size was also under selection. Thus, while consuming dairy products could have played a key role in promoting optimal pelvic shape, the fitness value of LP would relate more directly to promoting growth in overall pelvic dimensions and body size.

**TABLE 2:** Components of milk relevant to skeletal growth from humans and domesticated animal species.

Species	Energy^1^ (kJ/100 g)	Protein g/100 g	Calcium (mg/100 ml)	Phosphorus mg/100 g
Human	284	1.4	33	43
Horse	199	2.0	95	58
Donkey	156	1.6	91	61
Cow	263	3.4	113	84
Sheep	424	5.5	200	140
Goat	285	3.7	134	120
Buffalo	412	4.0	191	185
Dromedary	234	3.1	114	86
Bactrian camel	319	3.9	154	131
Llama	326	4.1	195	122
Alpaca	299	5.8	138	98
Yak	417	5.2	129	106
Mithun	510	6.5	88	147
Reindeer	819	10.4	320	270
Moose	538	10.5	280	276

*Values either point values, or the mid-point of ranges, assemble from references Barłowska et al. (2011); Medhammar et al. (2012), Claeys et al. (2014), and Martini et al. (2015).*

*^1^Most values approximated, using energy values for protein (17 kJ), lactose (16 kJ), and fat (37 kJ).*

#### Net Effect of Milk Consumption on the Obstetrical Dilemma

If consuming milk can promote the dimensions of both maternal pelvis and fetus, what might the net effects have been on childbirth complications? For several reasons, persistent milk consumption throughout development and adult life may have had greater effect on the pelvis. First, systematically drinking milk through childhood and adolescence would cumulatively have had a tangible effect on pelvic growth. Second, the calcium in milk may have reduced likelihood of pelvic deformities. Third, beneficial effects on pelvic development might have been explicitly realised by milk-consuming populations, with milk as a food prioritized for younger age groups. Conversely, it is likely that any tendency for milk to promote fetal growth would have been weaker. In populations that are not systematically overweight, the effect of consuming milk as an adult on maternal fatness may be relatively modest, and in women who are not overweight, maternal fatness is a much weaker predictor of fetal growth than is maternal lean mass (Wells, 2018b).

Regarding maternal milk consumption specifically during pregnancy, a review of data from high-income countries (where in every case the most common ethnic group comprised Europeans) found that consumption of cows’ milk was associated in some but not all studies with increased fetal growth and higher birth weight (Brantsæter et al., 2012). A number of underlying mechanisms may be involved, including increased maternal weight gain during the final trimester, enhanced placental growth, and maternal metabolic pathways including mTORC1 signalling and insulin resistance (Melnik et al., 2015). For example, observational studies from Denmark and India reported a positive association between milk intake in pregnancy and placental weight (Rao et al., 2001; Olsen et al., 2007).

In the Danish National birth cohort, consuming milk during pregnancy was associated with a relatively modestly increase in birth weights of 108 g, with a decreased risk of the neonate being small-for-gestational age, and an increased risk of it being large-for-gestational age (Olsen et al., 2007). Importantly, this greater birth weight led to greater adult height, showing how milk consumption could directly drive secular increases in body size (Hrolfsdottir et al., 2013). However, a systematic review found that the evidence for maternal milk intake and birth weight was heterogeneous, both for the presence/absence of such associations, and also their effect size (Brantsæter et al., 2012). The strongest effects were evident at the lower end of the milk consumption range. In India, for example, where the milk of both cows and water buffalo is consumed, maternal milk consumption during pregnancy was associated with birth weight in a rural cohort, but not in a smaller urban cohort (Rao et al., 2001; Wiley et al., 2016).

Overall, we suggest that the net effect of consuming animal milk through the life-course would be predicted to reduce the overall magnitude of the obstetrical dilemma, by promoting maternal height and pelvic shape through development and thus making childbirth less risky, even if the mother continued to consume milk during pregnancy itself, thus generating a modest increase in neonatal size.

## Selective Pressures on Lactase Persistence Deriving from Local Adaptation

Our overarching hypothesis is that LP could have undergone generic selection for its ability to systematically *reduce* the obstetric dilemma, even though the proximate factors *exacerbating* the obstetric dilemma may have varied locally. We argue that this benefit would have been primarily mediated by the impact of milk consumption on skeletal growth patterns in the life-course, in other words by impacting adult skeletal dimensions through plastic mechanisms rather than through genetic change. This plastic response would have *accelerated* the rate at which inter-generational trends in maternal size could have occurred, thus offering the potential to rapidly reduce the magnitude of the obstetric dilemma and hence reduce maternal mortality risk.

To achieve similar changes in maternal size through genetic change is expected to take much longer. The genetic basis of height variability is attributable to several hundred alleles, each exerting a relatively small magnitude of effect on adult height (Lango Allen et al., 2010). Thus, to achieve a substantial change in height through genetic change, a number of new polymorphisms would need to emerge, which would inevitably take time even in the context of rapid population growth (Cochran and Harpending, 2009). Very little is known specifically about the genetic basis of variability in human pelvic size and shape, though it appears to have a high degree of heritability (Sharma, 2002). However, studies of dogs, in whom selective breeding has led to substantial variability in pelvic size and shape, indicate a genetic basis for both components of phenotype, with some alleles associated with the IGF1 gene (Carrier et al., 2005; Fealey et al., 2017).

Before we consider the local ecological stresses that might have elevated selective pressure on LP, however, it is also worth noting that neutral evolution could also have exacerbated the obstetrical dilemma.

### Neutral Evolution

Some variation in human morphological dimensions may simply reflect neutral variation, which might itself pose challenges for the obstetric dilemma. A significant contribution of neutral variation to human variability has been reported for each of the pelvis (though less so for the obstetric pelvis) (Betti and Manica, 2018), cranial morphology (von Cramon-Taubadel, 2014), limb bone lengths (Roseman and Auerbach, 2015), and body mass (Speakman, 2008), though the extent to which this propagates to neonatal dimensions remains unknown. Neutral evolution represents an intriguing but under-researched issue for the obstetrical dilemma: beyond any effects of ecological stress or adaptation, neutral evolution could itself require ‘renegotiation’ of the fit between maternal and neonatal dimension.

While most neutral variation is geographically patterned (Ishida, 2009; Betti and Manica, 2018), we can understand its potential significance by considering the consequence of genetic variability within populations. Within populations, there is evidence of a degree of concordance within individual women between the physical dimensions of the head and the pelvis. Women with larger heads tend also to have birth canals with larger dimensions, while women of shorter stature have a pelvis characterized by a rounder inlet (Fischer and Mitteroecker, 2015). Such covariance has been proposed to indicate correlated selection between cranial and pelvic traits, and since head girth has high heritability, this indicates that maternal pelvic dimensions are more likely to match with fetal cranial dimensions (Fischer and Mitteroecker, 2015). Given such associations within populations, we assume that selection must also drive coordinated adaptive trends in the suite of traits relevant to the obstetric dilemma across geographical settings, in particular neonatal head size and the dimensions of the birth canal. Beyond any such neutral evolution, there are several other candidate selective pressures that could have impacted the obstetrical dilemma.

### Thermoregulation

Thermoregulation represents an intriguing potential selective pressure, for as we show below it may be relevant to the obstetrical dilemma in both cold and hot settings. In accordance with ecological ‘rules’ proposed by Bergmann and Allen (Bergmann, 1847; Allen, 1877), adult humans show an inverse association of body mass, and a direct association of limb length, with mean annual temperature (Roberts, 1953; Katzmarzyk and Leonard, 1998; Pomeroy et al., 2021). Both associations relate to thermoregulatory homeostasis: in colder settings, larger mass favours heat retention by reducing the area-mass ratio (AMR), while in hotter settings, longer limbs favour heat loss by increasing the AMR. The association for body mass holds for both its main components, fat-free and fat mass (Wells, 2012), with the association for lean mass particularly relevant as it is the primary determinant of fetal growth (Martin, 1983; Wells, 2018b).

If the body is treated as a cylinder, where its volume acts as a proxy for body mass, then increasing its length (equivalent to taller height) whilst holding its breadth constant changes body mass in isometric proportion, with no change in the AMR (Ruff, 1994). Conversely, increasing cylinder breadth (equivalent to more stocky physique) whilst holding its length constant, changes both mass and the AMR. From a thermodynamic perspective, variability in human body size is therefore expected to be targeted at stature for populations within a given thermal environment, but to be targeted at breadth for populations distributed across different thermal environments. Data on males and females support this hypothesis, showing a relatively constant bi-iliac breadth:stature ratio across a range of stature in populations from similar thermal environments, but increased bi-iliac-breadth:stature ratios at colder temperatures (Ruff, 2010; Wells et al., 2012).

**FIGURE 6:**
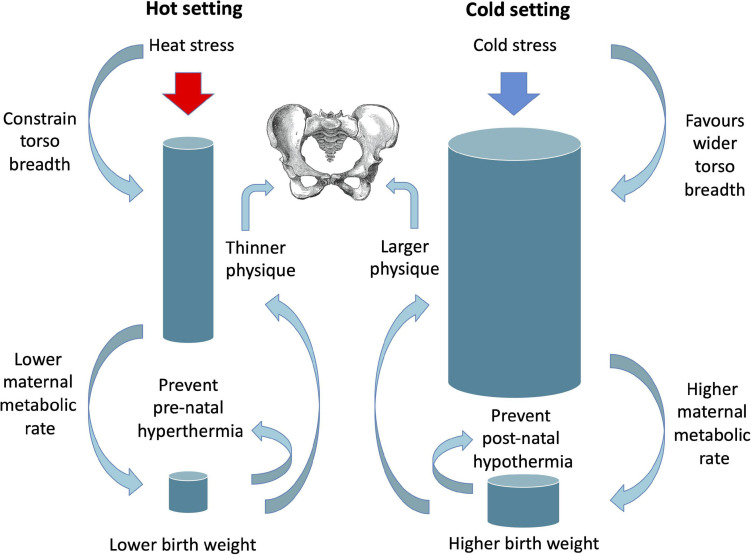
Schematic diagram illustrating potential selective pressures of different thermal environments on adult and birth size, with implications for pelvic size.

The significance of these associations for birth canal dimensions remain poorly understood, however a recent analysis reported a more oval canal in the transverse plane at higher latitudes (Betti and Manica, 2018). Such climate-associated pelvic variability is expected, given that the inverse association of body mass and temperature also holds for birth weight (Wells and Cole, 2002). As yet, which of adult versus neonatal phenotype is more important in this ecological association remains unknown (Figure 6). Higher adult mass and lean mass might be important to maintain adequate heat production among adults inhabiting cold settings, with the converse in hot conditions, and higher birth weight might be merely a contributing mechanism. Alternatively, higher birth weight might be strongly favoured in cold settings to prevent infant hypothermia, while lower birth weight in hot settings could reduce maternal heat stress during pregnancy (Wells, 2002), with variability in adult size merely a consequence of these relationships. Intuitively, both scenarios may be relevant, as thermal stress acts throughout the life-course.

Whatever the primary target of selection, delivering a larger neonate requires larger maternal pelvic dimensions. Hence, exposure to cold settings, mediated by selection on larger body size *sensu lato*, could have elicited selection on the capacity to consume milk through the life course. The consequences could include larger maternal fat-free mass and resting metabolic rate, greater fetal growth, and a larger birth canal through which to deliver the fetus. This perspective may help explain the rapid spread of LP in populations of farmers expanding northwards in Europe in the last 7000 years, approaching fixation in Scandinavian populations, but much less prevalent in southern European populations (Itan et al., 2010). In more recent historical periods, with improved living conditions, this could further explain substantial secular trends in female stature evident in northern but not southern European populations (Rosenstock et al., 2019).

In hot settings, selection favours lower overall body mass and narrower body breadth (Ruff, 1994), but in less humid environments there is greater potential to lose heat via skin surface area. Thus, in hot dry settings, greater length of the torso and limbs can promote cooling, consistent with empirical evidence on human morphology (Katzmarzyk and Leonard, 1998). Narrower body breadths and pelvic dimensions in hot settings must inevitably constrain neonatal size. Maternal thermodynamics may also impact fetal growth, as all fetal heat loss must occur via the maternal skin surface area (Wells, 2002). Greater fetal growth generates two impacts on maternal metabolism: greater energy transfer, and a correlated need for increased heat dissipation. Since these demands are antagonistic, greater fetal growth will inevitably increase maternal heat stress, thereby increasing the selective pressure on body proportions that promote heat loss. Although birth weight has a relatively weak genetic basis, aleles associated with low birth weight are disproportionately more common in African and Asian than European populations (Tekola-Ayele et al., 2018), suggesting ‘negotiation’ of reduced fetal growth in populations with narrower pelvic dimensions (Wells, 2015).

Again, therefore, the potential impact of milk consumption on maternal skeletal growth could promote fitness by tolerating higher birth weights, increasing neonatal survival. In this scenario, the benefits of LP could derive both from longer limb lengths promoting heat loss, and relatively greater pelvic dimensions (even if still narrow by global standards), which would help deliver the larger neonates.

This pathway may be especially relevant in the eastern African region, where significant climatic change occurred within the last 15,000 years (Kiage and Liu, 2006). Although until ∼42,000 BP the region had a warm climate similar to today, several rapid changes occurred subsequently. From 42,000–30,000 BP, cold dry conditions emerged, followed by cold moist conditions from 30,000–21,000 BP. Warmer and moister conditions re-emerged during the Holocene, followed by two major droughts, with the emergence of drier conditions from 4,000 BP. If these relatively rapid climate trends impacted human morphology, then the possibility for adaptation by direct genetic change in height may have been very limited. Accordingly, the recent emergence of hot dry conditions may have favoured the rapid spread of LP due to its capacity to impact growth traits through accelerated response to dietary factors.

On this basis, we suggest that the tall adult height of pastoralist populations may not necessarily have been selected because of its direct implications for adult subsistence activities, but rather for its contribution to renegotiating the obstetric dilemma and increasing maternal fitness in hot dry environments. Clearly, this ‘solution’ is only possible among populations that drink milk, and this may explain why adult size varies substantially by subsistence mode within East Africa (Galvin, 1992; Sellen, 1996; Pomeroy et al., 2021).

### Strengthening the Obstetric Pelvis in Low-UV Environments

In environments where the body experiences deficiencies of vitamin D and calcium, the consequence is that bone is built ‘on the cheap’ (Vieth, 2020). Incompletely mineralized bone is more flexible than highly mineralized bone, and this can lead to pelvic deformities. Even in the absence of any selection for larger body size in colder settings, low levels of vitamin D synthesis at higher latitudes could therefore have favoured increased dietary calcium intakes in order to improve bone mineralisation. This selective pressure is then expected to have been amplified, should selection for greater body size have co-occurred. In the absence of thickening of the bones, a wider pelvis is necessarily weaker, hence any increase in pelvic dimensions is expected to have increased dietary calcium demands.

Among human population entering environments with lower UV radiation levels, the selective pressure of rickets has specifically been proposed to have driven the evolution of lighter skin colour, thus increasing vitamin D synthesis despite lower total UV exposure (Vieth, 2020). Despite this, European populations show lower total bone quantity in adulthood compared to those of African ancestry, and are more prone to osteoporosis in later life. European populations may have evolved a profile of lower bone quantity, in order to maintain structural stiffness despite reduced calcium uptake. This would help prevent pelvic deformities by reducing the risk of softer, inadequately mineralised bone during the reproductive years, at a cost of more brittle bone quality in old age (Vieth, 2020).

Farming populations entering more northerly latitudes may therefore have faced not only cold stress but also, the selective pressure of supplying sufficient calcium to the developing skeleton and pelvis, to ensure viable childbirth. Since humans had already occupied northern Europe from ∼800,000 BP, the key stress is unlikely to have been cold or lack of sunlight *per se*, but rather the need for a *rapid adaptation* to these selective pressures as farmers pushed into more northerly habitats. Moreover, since farmers have higher fertility than hunter-gatherers, maternal calcium requirements might have increased, hence selection would have acted on the interaction between this greater mineral requirement and probing of northern latitudes. For example, low maternal calcium in breast milk increases risk of rickets in the offspring (Thacher et al., 2006).

On this basis, dairying itself would have been strongly favoured in early northern European farmers to increase calcium supply. Consistent with that, recent data indicate that the emergence of Neolithic farming beyond the 60th parallel north involved a transition at *∼*4500 BP from the exploitation of aquatic organisms to processing of ruminant products, specifically milk (Cramp et al., 2014a). Moreover, both mathematical models and archaeozoological and human skeletal evidence suggest that early farmers shifted very rapidly from exploiting marine resources to intensive dairy farming (Cramp et al., 2014b). Once dairying had been adopted, the combination of secular increases in body size and exposure to colder environments may then have favoured the rapid spread of LP alleles.

### Reversing Secular Declines in Stature Associated With the Origins of Agriculture

In any setting, the transition to agriculture may have challenged skeletal growth. Higher rates of protein intake predict taller adult stature, as evidenced by associations of vegan diets with shorter stature in contemporary children (Sanders, 1988; Desmond et al., 2021). Prior to farming, a higher proportion of dietary intake from hunting animals would likely have maintained higher stature, as supported by data on skeletal proportions from the archaeological record (Stock and Pinhasi, 2011). In contemporary populations, intake of animal protein remains correlated with global height variability (Grasgruber et al., 2016; Penuelas et al., 2017). The shift to crop agriculture may therefore have fundamentally challenged skeletal growth, along with broader changes to the subsistence niche.

In the 1980s, pioneering work showed that the transition to agriculture was widely associated with a decline in adult height, as well as an increase in markers of bone disease (Cohen and Armelagos, 1984). Subsequent studies have largely confirmed this finding, though there are occasional examples of no decline in height, or even a small short-term increase (Bogin and Keep, 1999; Gerhards, 2005; Mummert et al., 2011; Ozer et al., 2011; Pinhasi and Stock, 2011; Cohen and Crane-Kramer, 2012; Beresford-Jones et al., 2021). The emerging consensus is that declines in height reflect both dietary and ecological stresses (Mummert et al., 2011). First, early farming carried an increased risk of famine and food insecurity, while also providing a narrower range of nutrients. Second, more sedentary settlements also increased exposure to diverse infectious diseases, which are also likely to have inhibited linear growth (Urlacher et al., 2018). This is supported by evidence that declines in infant mortality rates (a marker of the infectious burden) are associated with secular increases in adult height (Crimmins and Finch, 2006). Beyond compelling evidence that infections are a key cause of child undernutrition and impaired growth (Vilcins et al., 2018; Schoenbuchner et al., 2019; Wells et al., 2019), vaccinations have been shown to improve growth (Anekwe and Kumar, 2012; Kim et al., 2017). A recent review suggested that the transition to agriculture elicited broader shifts in human life history strategy, with reduced energy allocation to maintenance and growth, and elevated investment in reproduction and defence (Wells and Stock, 2020).

We have argued previously that these height declines associated with the transition to agriculture would have had direct implications for childbirth (Wells et al., 2012). Any secular declines in height are predicted to have exacerbated the obstetric dilemma substantially, as the fetal head is relatively canalized across contemporary populations, and may have had less capacity to decrease in size across generations (Leary et al., 2006). Our hypothesis is supported by analyses from contemporary low- and middle-income countries, where short maternal stature increases the risk of caesarean section, and this effect is exacerbated by higher levels of body weight (Wells et al., 2018, 2020). This interactive association is important, as aside from constraining height, the transition to grain-based diets might, via their effects on insulin, have increased both maternal body fat levels and offspring birth weight (Wells et al., 2012).

For early European farmers, the potential benefits of greater linear growth and pelvic size could have substantially reduced maternal mortality, with more modest effects on the onset of the reproductive career, fertility rate and offspring survival. Interestingly, recent analysis has indicated major population crashes between ∼8000 and 4000 BP in Europe in the early agricultural era, which have not been linked with climatic stresses (Shennan et al., 2013), yet height also recovered from around 7000 BP in this region (Cox et al., 2021). Whether maternal mortality spiked due to the challenges posed by declines in height and lower levels of calcium and vitamin D merits further attention in this context.

Secular trends in height in the middle Eastern area remain poorly documented. Data from Anatolia suggest that from the Neolithic (12,000–7000 BP) to the Chalcolithic period (7000–5000 BP), female height declined by ∼3 cm, before recovering again by ∼4 cm during the Bronze age (5000–3000 BP) (Ozer et al., 2011). Changes in energy supply and the burden of disease associated with urbanization and increased state control of agriculture in this region (Graeber, 2011; Wells, 2016) could have driven such secular trends in height, at some stages exacerbating the obstetric dilemma. Once again, this could have favoured the spread of LP alleles and account for their contemporary elevated frequency in this region.

### Selection of Size Versus Growth

**FIGURE 7:**
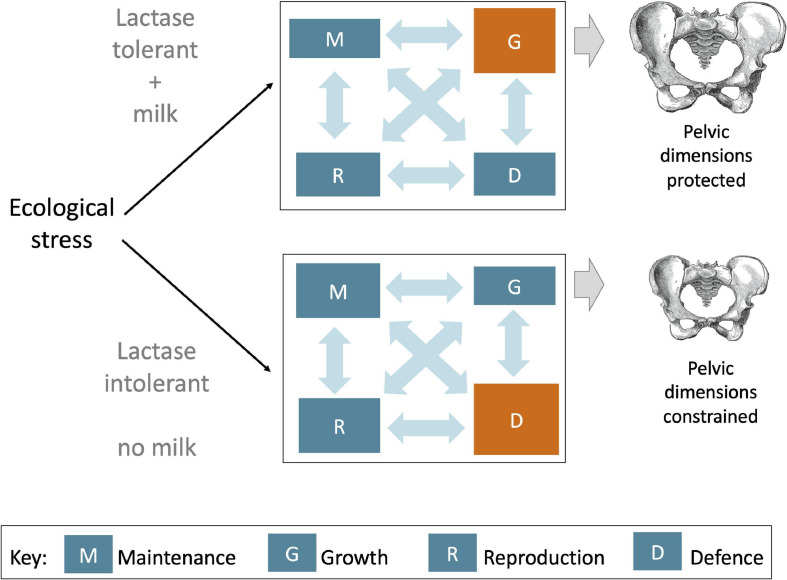
Schematic diagram illustrating how life history trade-offs could be affected differently during a period of ecological stress, depending on whether an individual is lactose tolerant or not. In general, ecological stress favours increased allocation of energy to the defence (immune defence and the stress response), at a cost to growth (lower panel). However, among lactose tolerant individuals consuming milk, more energy could be diverted to growth, potentially preserving pelvic dimensions despite lower overall energy supply, or by decreasing energy allocation to other life history functions.

Central to our argument is the notion that selection on LP alleles may have been driven not by variability in absolute height or size, but rather by the increased allocation of energy to growth traits given any specific genotype, diet and ecological setting. Among dairying populations, holding constant for overall genotype, energy/protein intake and any ecological constraints on growth, the presence of LP in an individual would specifically allow an increased allocation of energy to growth traits. In other words, LP would favour growth over other life history functions, through allowing the molecular components of milk to target growth-promoting mechanisms in a way not possible in the absence of LP, which would restrict dairy intakes to non-milk products (Figure 7). This ‘targeted’ impact of milk is, we suggest, what increased the selective pressure on LP.

### Mapping Human Growth Variability

While geographical variability in human height is well established (NCD Risk Factor Collaboration, 2016; Stulp and Barrett, 2016; Pomeroy et al., 2021), the underlying contribution of genetic variability remains surprisingly poorly understood (Stulp and Barrett, 2016). Moreover, secular trends in living conditions also contribute (Penuelas et al., 2017), making it difficult to evaluate the extent to which height itself has undergone positive selection. Recent analysis provides evidence for weak positive selection on height in northern compared to southern European populations (Turchin et al., 2012). This may be considered in the context of selection acting on reaction norms integrating trade-offs between growth, maintenance and reproduction (Walker et al., 2006; Wells and Stock, 2020).

**FIGURE 8:**
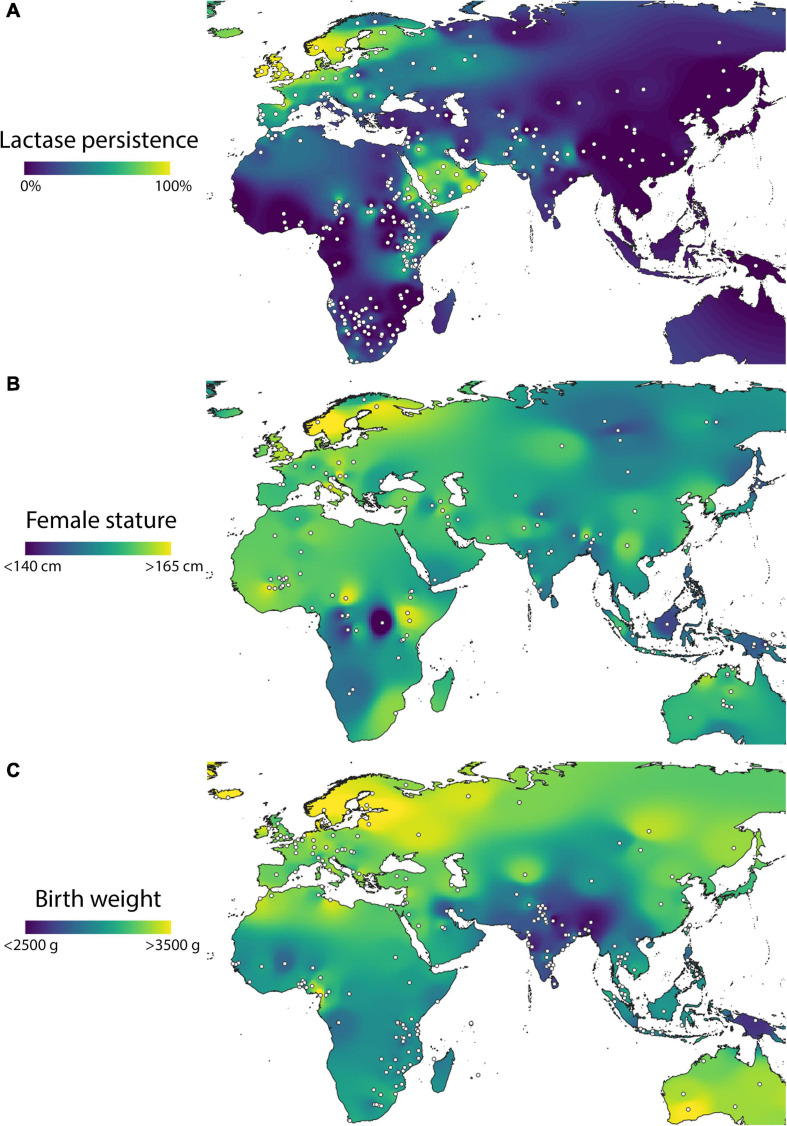
Geographic variability within the Old World of variability in **(A)** composite lactase persistence, **(B)** adult female height, and **(C)** birth weight. Data from Liebert et al. (2017) for lactase persistence, Pomeroy et al. (2021) for height, and Wells et al. (2016) for birth weight.

In this context, it is striking that contemporary maps of adult female height and LP show a correlated pattern. Height is systematically high in Europe but with a further positive gradient associated with northern latitudes, intermediate in northern Africa, and low in southern Africa and Asia, similar to data on LP prevalence. A similar pattern is discernible for birth weight, which again is systematically lower in southern Africa and Asia (Figure 8).

On this basis, we suggest that human height demonstrates substantial variability that does have a genetic basis, but one that is not necessarily only due to differences in individual polymorphisms associated with height variability. Instead, the single trait of LP might radically change the range of phenotype that an existing genotype can produce, mediated by milk consumption. Indeed, the remarkable secular trend in height in the Netherlands over the last 200 years appears to have involved little genetic change (Stulp et al., 2015), and to have primarily have involved changes in gene expression over multiple generations. This may therefore represent an interaction between past selection on LP, and recent changes in diet and living conditions.

More broadly, correlations between height and LP should not be taken as evidence that selection has acted directly on adult stature itself. More likely, the target of selection has been specific growth traits, that simply correlate with final stature. Moreover, human populations in close geographical proximity may show substantial contrasts in adult height, supporting the notion that this trait itself is not necessarily subject to strong positive selection (Stulp and Barrett, 2016). Again, such differences may have emerged through the mediating impact of milk consumption and LP on skeletal traits important for mortality or fertility.

## Growth in Populations Lacking Lactase Persistence

Our overarching hypothesis is that where human populations adopted dairying, ecological stresses impacting body size and growth could have generated strong selection on LP, because of the resulting relaxation of maternal mortality risk. Whereas other hypotheses have generally assumed selection to act directly on LP alleles due to some or other fitness pay-off, we assume that selection could only occur through the mediating role of variability in growth traits.

This scenario requires the combination of dairying, the emergence of mutations in the lactase gene that maintain activity of the enzyme, and appropriate selective pressures. Among non-dairying population, we predict that the speed with which growth traits could respond to ecological stresses, including new habitats reached through migration, would be much slower. Moreover, this would remain the case among dairying populations if no mutation in the lactase gene occurred, or if relevant selective pressures failed to manifest.

In this non-LP scenario, ecological constraints on growth are expected to have driven elevated maternal mortality associated with childbirth complications. There would therefore have been strong selective pressures on lower birth weight. For example, recent analysis of the archaeological record from the Indian subcontinent revealed evidence for the long-term persistence of slim physique over the last 10,000 years, which might reflect the consequences of neutral evolution in the early Holocene, as well as long-term downwards trends in height (Pomeroy et al., 2019). The Indian subcontinent is subject to major climate volatility, in association with regular El Niño events (Couper-Johnston, 2000). The threat of regular famines may counteract any benefits of growth traits being sensitive to short-term ecological improvements.

In contemporary South Asian populations, the relatively short stature and thin physique of adult women are associated with relatively small pelvic dimensions (Pan, 1929), which in turn constrain the range of birth weight (Wells et al., 2016). While the fetus may itself have a lower genetic potential for growth (Wells et al., 2013), possibly mediated by increased frequency of alleles for lower birth weight (Tekola-Ayele et al., 2018), behavioural practices such as ‘eating down’ in the last trimester in pregnancy may also be used deliberately to constrain fetal growth and thereby reduce the risk of childbirth complications (Choudhry, 1997). Where maternal BMI does increase in south Asian women, the likelihood of caesarean delivery and obstructed labour also rises, especially if the mother is also short (Wells et al., 2018, 2021). These patterns may also be relevant to other populations with smaller body size (Wells et al., 2020).

A notable pubic health paradox has been the limited secular increase in adult height in South Asia and sub-Saharan Africa (NCD Risk Factor Collaboration, 2016). Although there is a positive association between height and dietary intakes of animal-source protein (Penuelas et al., 2017), it is possible that populations with low LP frequencies cannot achieve the rapid secular increases in height seen in populations with high LP frequencies, such as northern European populations, or those in South America with high levels of European ancestry (NCD Risk Factor Collaboration, 2016). Conversely, former pastoralist populations who were habituated to consuming milk, but who have reduced their intake due to shifts in subsistence, experience high rates of growth faltering in children (Fratkin et al., 2004). This is consistent with evidence from high-income countries of children consuming milk-free diets (Tuokkola et al., 2017; Desmond et al., 2021).

While lower birth weights may reduce the risk of maternal mortality, they also have implications for health outcomes later in life. Most notably, in populations with low average birth weights, secular increases in adult BMI are leading to a high prevalence of type 2 diabetes and other non-communicable diseases (Wells et al., 2016, 2020; Miranda et al., 2019).

## Conclusion

In this article, we set out the novel hypothesis that LP alleles could have undergone rapid selection in different global regions under the unifying selective pressure of relaxing maternal mortality risk associated with obstructed labour. Since maternal mortality has major impact on inclusive fitness, it could have been a sufficiently strong selective pressure to have driven increases in the prevalence of LP in populations inhabiting very different ecological conditions.

Unlike most previous work on LP, our approach highlights the mediating role of growth variability. Our key argument is not that populations with LP are inherently taller, but that they *could have become taller relatively rapidly*, without changing overall dietary energy intake or the polygenic basis of height. This might have involved growing larger skeletal dimensions, but it could equally well have involved maintaining the same height even as ecological conditions worsened.

The emergence of a single genetic mutation could thus ‘turbo-charge’ adaptation in growth traits, in a way that would be impossible through conventional genetic change. We have offered examples of contrasting selective pressures in different geographical regions that could have favoured LP, in each case potentially reducing the ‘obstetric dilemma’. Our approach also emphasises human migration as a key mechanism through which populations may be exposed to ecological circumstances very different from those faced by their immediate ancestors.

Our perspective potentially sheds new light not only on the global distribution of LP, but also on contrasting rates of secular increase in body size. Rapid increases in height in some populations over the last two centuries have led to the notion that other populations have substantial height deficits (NCD Risk Factor Collaboration, 2020), but it remains unclear how rapidly these differences can resolve. This height variability is of major significance in medicine and public health, being associated with outcomes such as cardiovascular disease and diverse cancers (Paajanen et al., 2010; Green et al., 2011).

While the consumption of dairy products (rich in calories and calcium) does not require LP and is widespread, the variable emergence of LP over the last few millennia also has economic implications, as contemporary lactose-intolerant populations may miss out on the economic benefits associated with intense dairy agriculture. Thus, inequalities in LP contribute indirectly to contemporary economic inequalities, and the persistence of poverty in low-income countries. Ironically, such poverty may reduce the capacity of households in low-income settings to obtain nutritious foods for young children that promote optimal growth, including products that contain milk constituents or milk itself.

Our approach has some limitations. Although we have demonstrated evidence for a number of links in the causal chain, such as associations of maternal height with pelvic dimensions and the risk childbirth complications, or of milk consumption with linear growth, there is as yet minimal evidence for a direct link between milk consumption and maternal mortality risk, or between LP alleles and height variability. A series of outstanding questions are highlighted in Table 3. Historically, secular declines in infant mortality have been linked with subsequent secular increases in adult height (Crimmins and Finch, 2006), but whether maternal mortality also fell with increasing adult height is not yet known. Further work is needed to explore these issues.

**TABLE 3:** Issues that merit investigation in future work.

Mechanism
What is the association of milk consumption, measured prospectively in different developmental periods, with dimensions of the obstetric pelvis?
What is the association of metabolic signalling pathways involving milk constituents (e.g., IGF1, MTORC) with dimensions of the obstetric pelvis?
Contemporary epidemiology
What is the association of maternal height variability with the risk of dying in childbirth, allowing quantification of selective pressure on growth?
What is the association of maternal milk consumption with the risk of childbirth complications?
Paleo-epidemiology
What is the association of the LP allele with growth traits, holding constant for polygenic height allele scores, in the bio-archaeological record?
How do pelvic dimensions vary across time in populations characterized by changes in frequency of the LP allele?

## Data Availability Statement

Publicly available datasets were analyzed in this study. This data can be found here: The data are linked to published articles.

## Author Contributions

JW wrote the first draft. EP and JS provided critical feedback on revised versions. EP generated the maps. All authors conceived the manuscript, who were members of the UCL Diabetes Network.

## Conflict of Interest

The authors declare that the research was conducted in the absence of any commercial or financial relationships that could be construed as a potential conflict of interest.

## Publisher’s Note

All claims expressed in this article are solely those of the authors and do not necessarily represent those of their affiliated organizations, or those of the publisher, the editors and the reviewers. Any product that may be evaluated in this article, or claim that may be made by its manufacturer, is not guaranteed or endorsed by the publisher.
